# *Trypanosoma brucei* Invasion and T-Cell Infiltration of the Brain Parenchyma in Experimental Sleeping Sickness: Timing and Correlation with Functional Changes

**DOI:** 10.1371/journal.pntd.0005242

**Published:** 2016-12-21

**Authors:** Claudia Laperchia, Maria Palomba, Paul F. Seke Etet, Jean Rodgers, Barbara Bradley, Paul Montague, Gigliola Grassi-Zucconi, Peter G. E. Kennedy, Marina Bentivoglio

**Affiliations:** 1 Department of Neuroscience, Biomedicine and Movement Sciences, University of Verona, Verona, Italy; 2 Institute of Biodiversity Animal Health and Comparative Medicine, University of Glasgow, Glasgow, Scotland, United Kingdom; 3 Institute of Infection, Immunity and Inflammation, University of Glasgow, Glasgow, Scotland, United Kingdom; 4 National Institute of Neuroscience (INN), Verona Unit, Verona, Italy; Foundation for Innovative New Diagnostics (FIND), SWITZERLAND

## Abstract

**Background:**

The timing of *Trypanosoma brucei* entry into the brain parenchyma to initiate the second, meningoencephalitic stage of human African trypanosomiasis or sleeping sickness is currently debated and even parasite invasion of the neuropil has been recently questioned. Furthermore, the relationship between neurological features and disease stage are unclear, despite the important diagnostic and therapeutic implications.

**Methodology:**

Using a rat model of chronic *Trypanosoma brucei brucei* infection we determined the timing of parasite and T-cell neuropil infiltration and its correlation with functional changes. Parasite DNA was detected using trypanosome-specific PCR. Body weight and sleep structure alterations represented by sleep-onset rapid eye movement (SOREM) periods, reported in human and experimental African trypanosomiasis, were monitored. The presence of parasites, as well as CD4+ and CD8+ T-cells in the neuropil was assessed over time in the brain of the same animals by immunocytochemistry and quantitative analyses.

**Principal findings:**

Trypanosome DNA was present in the brain at day 6 post-infection and increased more than 15-fold by day 21. Parasites and T-cells were observed in the parenchyma from day 9 onwards. Parasites traversing blood vessel walls were observed in the hypothalamus and other brain regions. Body weight gain was reduced from day 7 onwards. SOREM episodes started in most cases early after infection, with an increase in number and duration after parasite neuroinvasion.

**Conclusion:**

These findings demonstrate invasion of the neuropil over time, after an initial interval, by parasites and lymphocytes crossing the blood-brain barrier, and show that neurological features can precede this event. The data thus challenge the current clinical and cerebrospinal fluid criteria of disease staging.

## Author Summary

Human African trypanosomiasis, or sleeping sickness, caused by *Trypanosoma brucei* infection, evolves from the first, haemolymphatic stage, to the second, meningoencephalitic stage. Classical knowledge sets the initiation of the second stage at the time of parasite invasion of the brain parenchyma; experimental findings have indicated that lymphocytes pave the way for this event. The disease leads to a complex neuropsychiatric syndrome, with sleep-wake alterations considered characteristic of the second stage. Staging criteria are still controversial and rely on cerebrospinal fluid examination. However, recent studies have questioned the route of parasite entry into the neuropil and even the occurrence of parasite neuroinvasion, as well as the correspondence of neurological signs and symptoms with the second stage of disease. In infected rats, we studied for the first time functional signs (body weight changes and sleep structure alterations) together with the presence of parasites and lymphocytes in the neuropil. The data show that parasites and lymphocytes invade the brain parenchyma over time, parasites cross blood vessels to enter the neuropil, and neurological features can precede these events. These findings question the cerebrospinal fluid criteria currently used for disease staging and focus attention on the importance of objective monitoring of clinical disease severity in sleeping sickness.

## Introduction

Human African trypanosomiasis (HAT) or sleeping sickness is a severe neglected tropical disease caused by the protozoan parasites *Trypanosoma brucei* (*T*. *b*.*)*, spread by tsetse fly vectors (genus *Glossina*). The disease is endemic in foci in sub-Saharan Africa [1, 2]. After a peak of infection in the 1990s, the incidence of HAT has considerably declined in recent years [1]. It is estimated, however, that a significant number of new cases remain unreported or undiagnosed [3–5]. It is also important to consider that there is a lengthy lag in outbreaks of the disease after periods of conflict or socio-political instability [6]. Concern is currently raised by outbreaks in the Ivory Coast [7] and South Sudan [8], by the discovery of the presence of asymptomatic carriers [9, 10], and by the resurgence of an old focus in Zambia [11].

The *T*. *b*. *gambiense* subspecies causes the Gambian or West African form, with a chronic clinical course, representing the vast majority of cases, and *T*. *b*. *rhodesiense* is responsible for the more acute Rhodesian or East African form of HAT [5]. The *T*. *b*. *brucei* subspecies causes disease in animals but not in humans and is widely used in rodent models of the infection [12].

HAT evolves in two stages leading to a complex neuropsychiatric syndrome dominated by sleep alterations. Experimental findings have shown that in the first, haemolymphatic stage, parasites proliferate in the blood and lymphatics and invade peripheral organs. In the brain, they reside in the choroid plexus and circumventricular organs, in which the blood-brain barrier (BBB) is highly permeable [13]. It is classically considered that invasion of the central nervous system (CNS) by African trypanosomes initiates the second, meningoencephalitic, stage [4, 5, 12] which is almost always fatal if left untreated.

Accurate disease staging is crucial for treatment as the drugs used to cure the first stage cross the BBB inefficiently and cannot cure CNS-stage disease, while drugs used for second stage treatment are very toxic [5]. According to WHO recommendations [14], the second stage of HAT is defined by the presence of trypanosomes and/or increased white blood cell count in the cerebrospinal fluid (CSF). However, the exact values ascribed to these criteria and their ability to accurately define disease stage are highly debated [4, 5, 15–17].

Experimental studies have indicated that parasite neuroinvasion occurs by transmigration of the BBB, and that lymphocyte recruitment to the brain paves the way to this event [12]. However, routes and mechanisms used by the parasite to gain entry into the brain remain to be fully clarified [18–20]. The timing of parasite neuroinvasion following infection is also controversial [21, 22], and even parasite entry into the brain parenchyma has been recently questioned [19, 20, 23]. Importantly, neurological signs and symptoms are considered characteristic of the second stage of HAT, but their onset and evolution in relation to the disease second stage, which have obvious diagnostic and therapeutic implications, remain to be determined [5].

Body weight changes, frequently found in HAT patients [15, 24], have been proposed as a sign of second stage disease in rats following *T*. *b*. *brucei* infection [25, 26]. The sleep-wake disorder, which gave the disease its alternative name of sleeping sickness, leads to sleep-wake cycle disruption and alterations in sleep architecture represented by sleep-onset rapid eye movement (SOREM) periods [27, 28]. In these episodes, the normal structure of sleep, represented by cycles of slow wave sleep epochs followed by rapid eye movement (REM) sleep [29], is altered, so that REM sleep is preceded by wakefulness [30, 31]. The onset of SOREM periods has been previously considered a robust sign of second stage disease in *T*. *b*. *brucei*-infected rats [32], and suggested as a marker of the second stage of HAT [27]. However, the correspondence between the clinical onset of neurological features and the second stage of the disease has been recently questioned [5, 24], and neurological signs can occur early in *T*. *b*. *rhodesiense* infections [33, 34].

Using a rat model, we here determined both the level and timing of parasite and T-cell entry into the brain parenchyma, thus testing the hypothesis that the onset of neurological alterations, which could represent markers of second stage disease, may not correlate with this event. To this purpose, parasite load in the brain was investigated over time. Furthermore, two functional parameters, body weight and sleep structure alterations, were monitored, and the presence of trypanosomes and T-cells in the neuropil was assessed in the same brains during the progression of the infection.

## Materials and Methods

### Ethics statement

The experimental protocol received approval by the Animal Care and Use Committee of the University of Verona and authorization by the Italian Ministry of Health (protocol n°18/2012-B). The experiments were conducted under veterinarian assistance, according to the European Communities Council (86/609/EEC) directives and ARRIVE guidelines.

### Animals and infection

Young adult male Sprague–Dawley rats (Charles River, Calco, Italy), weighing 200–250 g pre-infection, were used. The animals were given food and water *ad libitum* and kept under controlled temperature and humidity, with a 12 h:12 h dark-light cycle, for at least three weeks prior to the experiment.

The rats were infected i.p. with *T*. *b*. *brucei* AnTat 1/1E, a pleiomorphic clone derived from stabilate EATRO (East African Trypanosomiasis Research Organization) 1125 (kindly supplied by the Laboratory of Serology, Institute of Tropical Medicine, Antwerp, Belgium). At 3 days post-infection (dpi), blood samples were collected from the tip of the tail and examined under a microscope to verify parasitaemia. The intradermal route of infection has also been used in previous studies in mice, but higher sensitivity to i.p. injection was found [35]. We here used a well standardized model of i.p. infection of rats which causes chronic disease, with an average duration of 35 days [36].

### Experimental design

The infected rats were randomly assigned to three experimental cohorts. The first group was used to determine trypanosome load in the brain. The rats were sacrificed at 6, 14 and 21 dpi (*n* = 3 per time point), and matched with uninfected rats (*n* = 3). Under deep anesthesia, animals from group 1 were transcardially perfused with saline to remove the blood from the vasculature, and the brains were excised and frozen for subsequent DNA extraction.

Animals of groups 2 and 3 were deeply anesthetized and sacrificed by cervical dislocation. The second group was used to monitor body weight and study the temporal and regional features of parasite neuroinvasion and T-cell recruitment into the brain. Body weight was recorded at 10–11 am daily, beginning prior to infection until the endpoint of the experiment. The animals were sacrificed at 4, 9, 12, 15, 18, 21 and 24 dpi (*n* = 3 per time point). Uninfected rats (*n* = 3) were also culled to act as controls. At sacrifice the brain was excised and processed for histopathological analyses. The quantitative analysis of immunolabeled parasites and lymphocytes was performed in a blinded fashion so that the assessors were unaware of the infection stage of the animals.

The third experimental group was set up to investigate the relationship between sleep structure alterations represented by SOREM periods and parasite invasion of the brain parenchyma. These animals were subjected to telemetric recording and sacrificed at 11 (*n* = 1), 15 (*n* = 2), 19 (*n* = 1) and 21 (*n* = 1) dpi. Quantitative analyses of the number of immunolabeled parasites in the neuropil were performed in the brain of these animals after sacrifice. The analysis was conducted blindly of the animal’s electroencephalography (EEG) data and time of sacrifice.

### DNA extraction and quantitative PCR

Trypanosome load in the brain was determined using Taqman real-time PCR. Under deep anesthesia (pentobarbital, 50 mg/Kg, i.p.), animals from the first group were perfused transcardially with 120 ml of sterile saline to wash out the blood. The brains were extracted, immediately frozen on solid carbon dioxide and stored at -70°C until required. DNA was prepared from a 25 mg sample of whole brain homogenate following proteinase K digestion (DNeasy Tissue kit; Qiagen, Manchester, UK). Taqman PCR, using primers and probe specifically designed to detect the trypanosome *Pfr*2 gene, was performed in a 25 μL reaction mix comprising 1 x Taqman Brilliant II master mix (Agilent, Stockport, UK), 0.05 pmol/μL forward primer (ccaaccgtgtgtttcctcct), 0.05 pmol/μL reverse primer (gaaaaggtgtcaaactactgccg), 0.1 pmol/μL probe (fam-cttgtcttctccttttttgtctctttccccct-tamra) (Eurofins MWG Operon, Ebersberg, Germany) and 100 ng template DNA. A standard curve was constructed using a serial dilution (range; 1 x 10^6^ to 1 x 10^1^ copies) of pCR^®^2.1 vector containing the cloned *Pfr*2 target sequence (Eurofins MWG Operon). The amplification was performed on a MxPro 3005 (Agilent) with a thermal profile of 95°C for 10 minutes followed by 45 cycles of 95°C for 15 seconds, 60°C for 1 minute and 72°C for 1 second. The trypanosome load within the brain samples was calculated from the standard curve using the MxPro qPCR software (Agilent).

### Telemetric recording

In rats of the third group, used to investigate the relationship between sleep alterations and parasite neuroinvasion, sleep and wake were continuously monitored with telemetric recording. Telemetric probes (TL11M2-F40-EET, Data Science International, Arden Hills, MN, USA) were chronically implanted under deep anaesthesia (tiletamine-zolazepam hydrochloride, Zoletil, Virbac Lab, Carros, France; 20 mg/kg, i.p.). A probe was implanted in the abdominal cavity and four electrodes were tunnelled subcutaneously to the animal’s head. The first pair of electrodes was placed epidurally on the right side of the skull (frontal electrode 2 mm anterior to bregma and 2 mm lateral to the midline, parietal electrode 4 mm anterior to lambda and 2 mm lateral to the midline) and fixed in place with dental acrylic cement to allow EEG signal detection. The remaining two electrodes were inserted in the neck muscles for electromyography (EMG) signal detection. The incisions were then sutured, and the animals received analgesic (Carprofen, 5 mg/kg s.c.; Rimadyl, Pfizer, Karlsruhe, Germany), as well as antibiotic (Clamoxyl, 150 mg/kg s.c; Beecham Lab, Brentford, UK) treatment. The animals were then placed in the recording chamber and allowed to recover for 10 days. After two days of baseline recordings, the animals were infected with *T*. *b*. *brucei* as described above. The telemetry device continuously received, processed and transmitted data from the data storing remote receiver and computer-based running data (Dataquest IV, Data Sciences, St-Paul, MN, USA).

### Sleep and wake analysis

Using MATLAB software (MathWorks, Natick, MA, USA), trained observers visually scored EEG and EMG signals for the determination of sleep-wake states: wakefulness, characterized by low intensity and high frequency of EEG, concomitantly with relatively high EMG intensity; slow wave sleep, characterized by high intensity and low frequency of EEG, concomitantly with lower EMG intensity; REM sleep, characterized by low intensity and high EEG frequency, concomitantly with very low EMG intensity. SOREM episodes were scored as events lasting at least 5 seconds marked by REM sleep-like EEG tracing and a sudden drop of EMG intensity, preceded by more than 30 seconds of uninterrupted wakefulness [37]. The number and duration of SOREM episodes were calculated over 12 h corresponding to light and dark phases, respectively, and expressed as individual values.

### Brain tissue processing and immunofluorescence

The rats of the second and third experimental groups were deeply anesthetized (tribromoethanol in 2-methyl-2 butanol; 20 mg/Kg ip), sacrificed by cervical dislocation, and the brains quickly removed, frozen on crushed dry ice, and stored at –80°C until processing. Frozen brains were then cut on a cryostat into 20 μm-thick sections which were mounted on chrome-alum gelatin-coated slides, air dried, and stored at -20°C until use. Prior to use, the sections were fixed in 4% paraformaldehyde in 0.01M phosphate-buffered saline, pH 7.4 (PBS) at 4°C for 30 seconds, rinsed in PBS, fixed in acetone at –20°C for 2 minutes and rinsed again in PBS.

Brain sections from the rats of the second experimental group (in which body weight was monitored) were destined for double immunofluorescent labeling of blood vessel endothelia and *T*. *b*. *brucei*, or blood vessel endothelia and CD4+/CD8+ T-cells, respectively. Brain sections from rats of the third experimental group (which were monitored with telemetric recording) were earmarked for double immunofluorescent labeling of blood vessel endothelia and *T*. *b*. *brucei*.

In both these experimental groups, brain sections were selected (in groups of 4 adjacent sections per level and per animal) at 2 anteroposterior levels: an anterior level passing through the septum (-0.11 from bregma) [38] and a posterior level passing through the diencephalon at the level of the median eminence (-1.08 from bregma). Sections from each of these two levels were stained with cresyl violet for cytoarchitectonic reference, and the three consecutive sections were processed for immunofluorescence to visualize the parasites, CD4+ and CD8+ T cells, respectively.

#### Double immunofluorescence

Glucose transporter-1 (Glut-1) was used as a marker for endothelial cells in cerebral microvessels [39]. The sections were initially pre-incubated in a solution of 5% bovine serum albumin and 0.3% Triton X-100 in 0.1M phosphate buffer. The same solution was used to dilute primary and secondary antibodies in the subsequent steps of the procedure. After pre-incubation, the sections were incubated for 24 h at 4°C in a mixture of primary antibodies: goat polyclonal anti-Glut-1 antibodies (dilution 1:100; Santa Cruz Biotechnology, Santa Cruz, CA, USA), and one of the following primary antibodies: *i*) polyclonal mouse anti-rat CD4+ (dilution 1:100; Becton Dickinson, Buccinasco, Milan, Italy); *ii*) polyclonal mouse anti-rat CD8+ (dilution 1:100; Becton Dickinson); *iii*) rabbit polyclonal antibodies which recognize the variant surface glycoprotein of the AnTat 1:1E stabilate (dilution 1:500; kindly supplied by the Institute of Tropical Medicine, Antwerp, Belgium). The sections were rinsed in PBS and incubated in a solution of secondary antibodies containing Alexa Fluor 488-conjugated donkey anti-goat IgGs, and Alexa Fluor 546-conjugated donkey anti-mouse IgGs or Alexa Fluor 488-conjugated donkey anti-rabbit IgGs. All secondary antibodies were purchased from InVitrogen Corporation (Carlsbad, CA, USA) and used at a 1:200 dilution. The sections were rinsed in PBS, mounted using a fluorescence-compatible medium (Dako, Hamburg, Germany) and stored at 4°C.

### Microscopy and cell counts

The sections were analysed by confocal microscopy (Zeiss LSM 510 Carl Zeiss, Jena, Germany). For 3D reconstruction, confocal images were processed with the Macintosh version of the Adobe Photoshop CS6 and Adobe Illustrator CS5.1 softwares (Adobe Systems Inc., San José, CA, USA).

Quantitative analyses were performed in different regions of each brain using a semi-automated computerized system comprising a Nikon Eclipse 600 fluorescence microscope. Anatomical subdivisions (delineated using the corresponding Nissl sections) were first identified at low power magnification (10X objective) and defined landmarks established. Each area was then scanned at higher magnification (40X and 63X objectives). Pictures were acquired by a digital camera, deblurred using advanced image deconvolution functions of Autoquant X v2.2 software (Media Cybernetics Inc., Bethesda, MD, USA), and using the Colour Composite module and the Co-Location function of the Image-Pro Plus v4.5 software (Media Cybernetics Inc.). The immunolabelled *T*. *b*. *brucei* or T-cells found outside the blood vessels within the neuropil were counted bilaterally by screening along the X and Y axes in regular steps (40X objective, frame size: 230.34 X 230.34 μm; 63X objective, frame size: 146.25 X 146.25 μm), as previously described [40]. Cell counts were expressed as individual values: number of *T*. *b*. *brucei*, CD4+ cells, CD8+ cells in the brain parenchyma of each rat.

### Statistical analyses

The qPCR data was analysed using analysis of variance (ANOVA; general linear model) followed by Tukey’s *post-hoc* test in Minitab. Body weight differences were analysed with ANOVA mixed linear model (time x treatment) followed by multiple pairwise Bonferroni *post-hoc* test (SPSS v.24 software, IBM Corporation, Armonk, NY, USA). Cell counts were analysed with the Wilcoxon paired non-parametric test. For all analyses, significance threshold was set at the 95% level. Correlation analyses were performed using the non-parametric Spearman correlation test (GraphPad Prism 5 software, GraphPad, La Jolla, CA, USA).

## Results

### Timing and progression of *T*. *b*. *brucei* invasion and T-cell infiltration of the brain parenchyma

**Fig 1:**
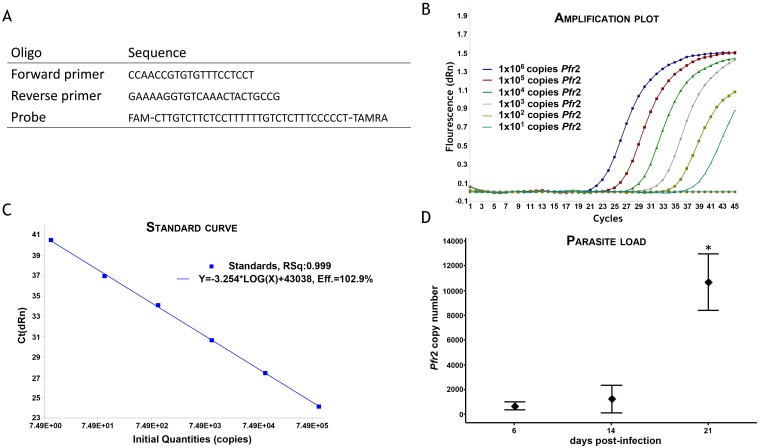
Taqman PCR for determining parasite load. **A:** Primer and probe sequences used in Taqman PCR to detect and quantify *T*. *b*. *brucei Pfr2* DNA. The amplification plot **(B)** and standard curve **(C)** obtained using a 10 fold dilution of standards containing 1x10^6^ to 1x10^1^ copies of the *Pfr2* gene sequence indicate that the assay performs with high efficiency (102.9%) and returns a linear response (R^2^ = 0.999) across a wide range of template concentrations. **D:** The interval plot represents the trypanosome load measured using this Taqman PCR assay within rat brains at 6, 14 and 21 day post-infection (dpi), *n* = 3 at each time-point; ♦ indicates the group mean; bars represent 95% confidence interval for the group mean; * significantly higher (P<0.001) than unmarked groups.

Parasite DNA in brain tissue homogenate (Fig 1) was detected at 6 dpi (659.8 ± 74.4 copies of *Pfr*2). Parasite load in the brain almost doubled at 14 dpi (1219 ± 265) although this rise was not significant. Trypanosome load in brain tissue at 21 dpi (10,660 ± 531) was significantly higher (P<0.001) than at either of the two earlier time points.

**Fig 2:**
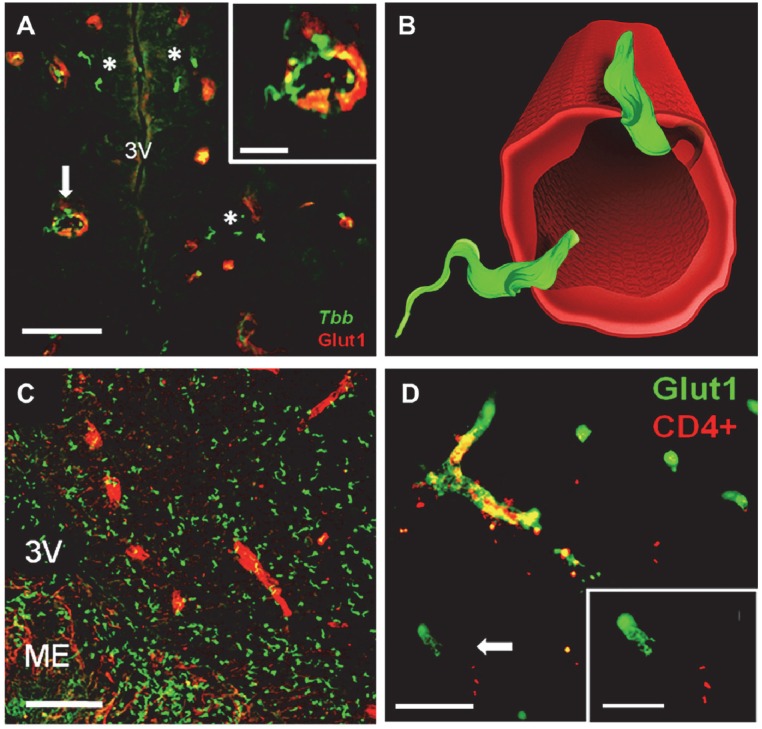
Confocal microscopy images of *T*. *b*. *brucei* (*Tbb*) and T-cell infiltration of the neuropil. Blood vessel endothelia are visualized by glucose transporter-1 (Glut1) immunolabeling. **A:** *Tbb* (asterisks) in the neuropil outside blood vessels in the hypothalamus (3V, third ventricle) at 9 day post-infection (dpi). The inset in **A** show at higher magnification the images indicated by the arrow, with parasites on the blood vessel wall and traversing it. **B:** 3-D reconstruction of parasites crossing a blood vessel shown in the inset on the top in A; note the different orientation of the flagellum of the two parasites, which suggests bidirectional transmigration. **C:** *Tbb* invasion of the neuropil of the posterior hypothalamus at 21 dpi; note the concentration in the median eminence (ME), a circumventricular organ in which the blood-brain barrier is highly permeable. **D:** CD4+ T-cells infiltrating the brain parenchyma at 21 dpi. The inset shows at higher magnification the area indicated by arrow, an example of neuropil in which T-cells are found outside blood vessels. Scale bars: A,C,D: 20 μm; insets in A,D: 10 μm.

**Fig 3:**
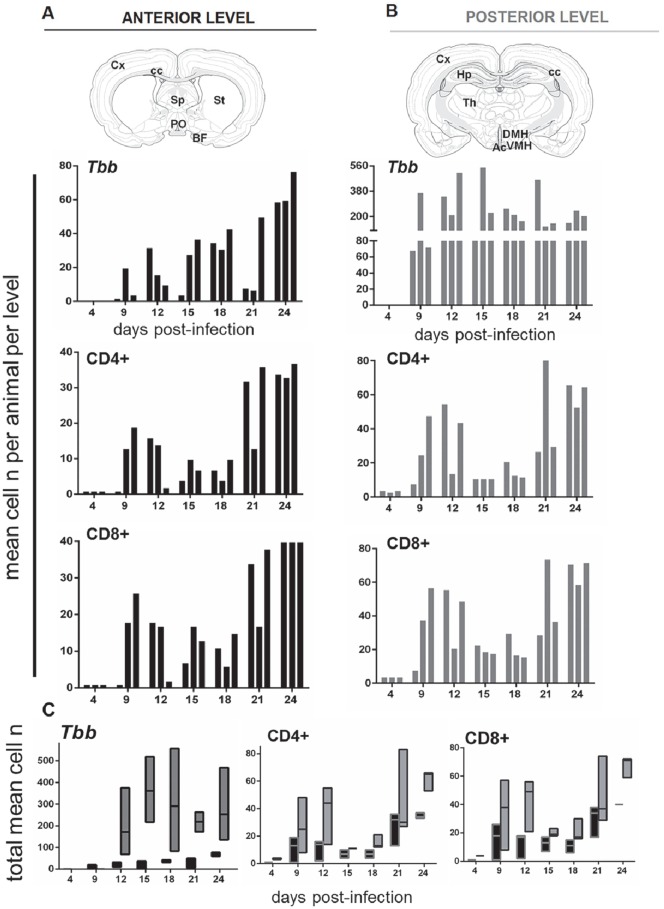
Quantitative analyses of the infiltration of parasites and T-cells in the brain parenchyma at different days post-infection. **A,B:** Brain sections from the rat brain stereotaxic atlas [38] represent the anterior and posterior levels and structures selected for the analysis and counts (n, number) of parasites (*T*. *b*. *brucei*, *Tbb*) and T-cells (CD4+, CD8+), respectively, performed in three adjacent sections through the anterior level (black columns) or at the posterior level (gray columns) in three rats sacrificed during the progression of the infection. Each bar corresponds to one animal, and counts in the same animal are presented vertically in each column at anterior and posterior levels, respectively. **C:** floating bars show the range (from minimum to maximum values; horizontal bar indicates the median) of the numbers of *Tbb*, CD4+ and CD8+ cells at the anterior (black columns) and posterior (gray columns) levels. Abbreviations: Ac, arcuate nucleus; BF, basal forebrain; DMH, dorsomedial hypothalamus; cc, corpus callosum; Cx, neocortex; Hp, hippocampus; PO, preoptic area; Sp, septum; St, striatum; Th, thalamus; VMH, ventromedial hypothalamus.

Double immunofluorescence allowed simultaneous detection of parasites and blood vessel walls (Fig 2A and 2C). The parasites were seen within blood vessels at 4 dpi and from 9 dpi onward also in the neuropil (Fig 3). Interestingly, parasites traversing blood vessel walls were observed in the hypothalamus, with an orientation of the flagellum which suggested a bidirectional transmigration (Fig 2A and 2B). Parasites traversing blood vessel walls were also seen in other brain regions, e.g. in the cerebral cortex. Trypanosomes were witnessed sporadically within the third ventricle, but parasite aggregates along the ependymal lining of the ventricles were never observed (Fig 2A and 2C). A variable number of parasites were observed in the choroid plexus.

**Fig 4:**
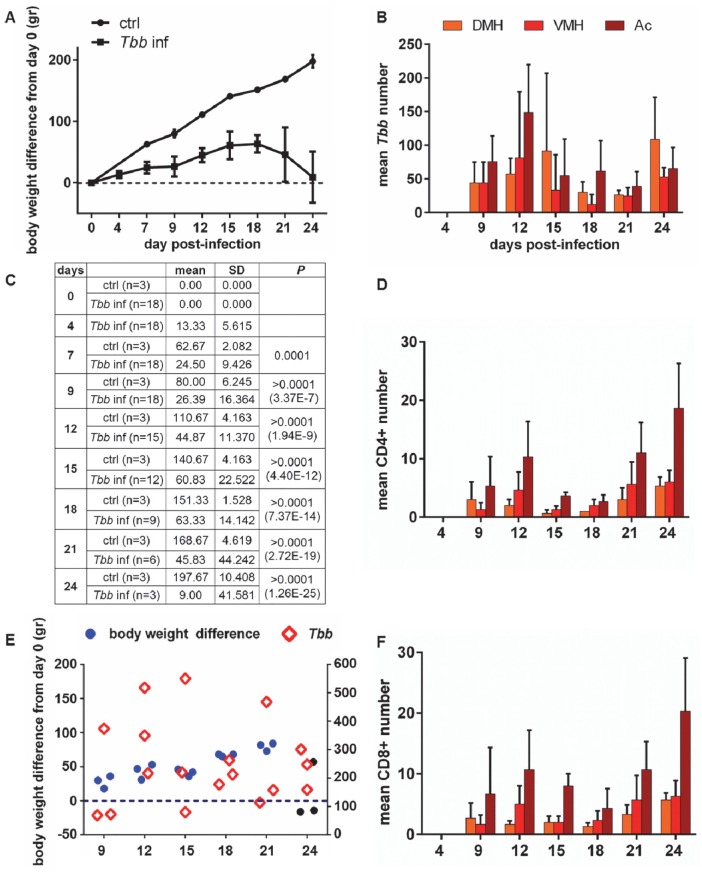
Body weight changes during the course of the infection and quantitative analysis of parasites and T cells in the brain parenchyma. **A:** body weight gain difference (mean values ± SD) monitored in the groups of rats (see C for sample size) from the day before *T*. *b*. *brucei* (*Tbb*) infection (day 0) until 24 day post-infection (dpi), compared with uninfected rats (ctrl, control). Two-factor (time x infection) mixed-design ANOVA for repeated measures showed a significant main effect both for infection (F_1,26_ = 224.26, p<0.001) and time (F_8,90_ = 40.19, P<0.001). **B**,**D**,**F**: Counts (mean number + SD in three rats per time point) of *Tbb*, CD4+ and CD8+ T cells, respectively, in adjacent sections through hypothalamic centers which play a key role in the regulation of appetite: DMH: dorsomedial hypothalamus. VMH: ventromedial hypothalamus. Ac: arcuate nucleus. **C**: the table shows the sample size (groups of 3 rats were sacrificed at different days) and summarizes the statistical analysis of body weight difference between uninfected controls and infected rats during the progression of infection, based on Bonferroni *post-hoc* test following ANOVA. **E:** scatter plots of the individual values of body weight difference from day 0 (left Y axis; blue and black dots) and total number of parasites counted in the brain parenchyma of each rat (right Y axis; diamonds) in the groups of three rats sacrificed during the progression of the infection; the black dots correspond to the 3 animals of the last group, sacrificed at 24 dpi (see A and C).

The comparison of the number of parasites detected within the neuropil in anterior *versus* posterior levels during the infection is shown in Fig 3 and parasite regional prevalence in the Supporting Information S1 Fig. From 9 dpi onwards, the number of parasites in the neuropil was approximately 7-fold higher posteriorly (Fig 3B) than anteriorly (Fig 3A), though with high inter-individual variability at both levels. A prevalence of parasites at the posterior level *versus* the anterior level was observed at all time points (Fig 3C), although the difference did not reach statistical significance. At the anterior level, the number of parasites in the neuropil showed considerable inter-individual variability between 9 and 21 dpi, and was relatively high in all animals at 24 dpi (Fig 3A and 3C). This trend was not evident at the posterior level, in which the number of parasites was consistently elevated from 9 dpi onward (Fig 3B and 3C). Overall, in terms of temporal sequence, the findings point to the end of the first week and second week post-infection as a critical period for parasite entry into the parenchyma (Figs 3 and 4B; Supporting Information S1 Fig).

Blood vessel walls together with CD4+ or CD8+ T-cells were also visualised by double immunofluorescence, thus allowing localization of lymphocytes within the vessels or in the neuropil (Fig 2D). Of particular interest is the comparison of the temporal progression and regional prevalence of lymphocyte infiltration with parasite neuroinvasion (Fig 3, Supporting Information S1 Fig). Isolated CD4+ and CD8+ T-cells were seen in the neuropil at 4 dpi, and were more frequently detected in the neuropil at 9 dpi in two of the three cases, and in all cases from 12 dpi onwards. The number of each T-cell population in the neuropil varied during the progression of the infection at both anterior and posterior levels (Fig 3A–3C), and in most instances the number of CD4+ T-cells and CD8+ T-cells was approximately 2-fold higher at the posterior than at the anterior level (Fig 3C). Interestingly, the total number of lymphocytes infiltrated into the brain parenchyma positively correlated with the number of infiltrated parasites over time (Spearman correlation: *T*.*b*. *vs* CD4+ cells, r = 0.821; *T*.*b*. *vs* CD8+ cells, r = 0.751).

Regional parasite distribution confirmed the infiltration of trypanosomes anteriorly in the neocortex, septum, corpus callosum, striatum and basal regions (basal forebrain and medial preoptic area) (Supporting Information S1 Fig), with relatively high numbers of parasites in hypothalamic regions at the posterior level (Fig 4B). Parasites were also observed at the posterior level in more dorsal regions of the diencephalon, i.e. the thalamus and the telencephalon (hippocampus, corpus callosum, neocortex) (Supporting Information S1 Fig). In the hypothalamus the structures exhibiting the most marked parasite invasion were the arcuate nucleus and dorsomedial hypothalamus (Fig 4B), which, together with the ventromedial hypothalamus, are part of the feeding-regulatory neural network [41].

CD4+ T-cells and CD8+ T-cells grossly exhibited features similar to those of parasites in terms of regional distribution, with a prevalence in the basal forebrain and preoptic area at the anterior level and a concentration at the posterior level in the hypothalamus from 9 dpi onward (Fig 4D and 4F; Supporting Information S1 Fig).

### Body weight and *T*. *b*. *brucei* neuroinvasion

Two-factor (time x infection) mixed-design ANOVA for repeated measures showed a significant main effect both of the infection (F_1,26_ = 224.26, p<0.001) and time (F_8,90_ = 40.19, P<0.001) on body weight. In particular, a significant body weight gain (p<0.001) was observed over time in uninfected rats during the period of monitoring (24 days). The infected rats did not show significant body weight gain in the same period. *Post-hoc* testing showed that body weight gain was significantly lower in the infected rats with respect to uninfected controls from 7 dpi onwards (P<0.0001 at 7–24 dpi; Fig 4A and 4C).

No direct relationship was found between decrease of body weight gain and total number of parasites in the neuropil at the analysed levels, which showed at all time points a great inter-individual variability (Fig 4E), as described above (Fig 3).

### SOREM periods and *T*. *b*. *brucei* neuroinvasion

**Fig 5:**
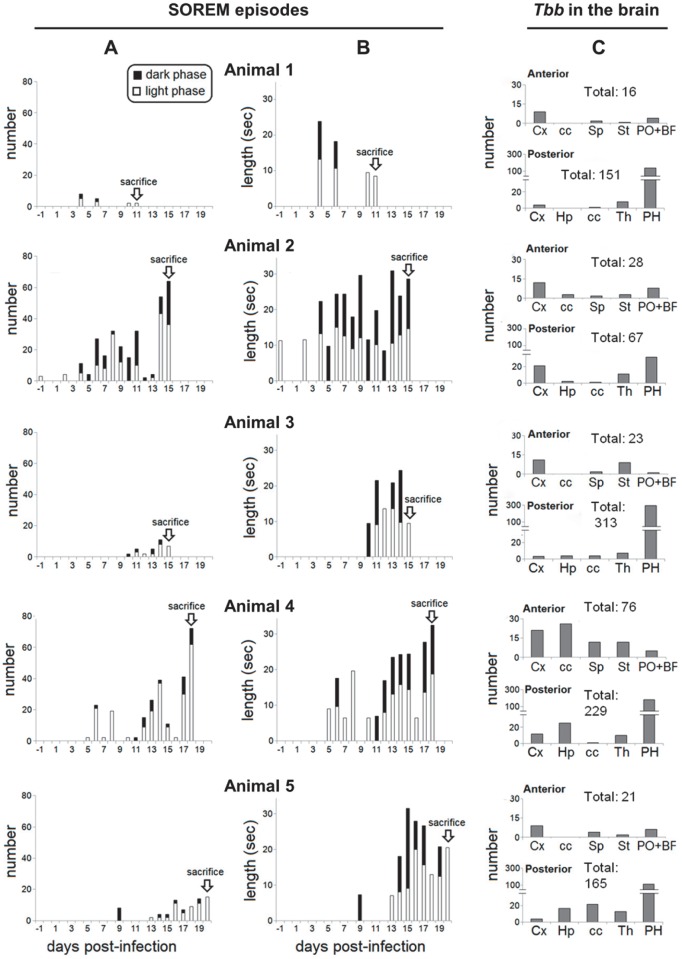
SOREM episodes during the course of the infection and quantitative analysis of parasites in the brain parenchyma. The number (first column, **A**) and duration (second column, **B**) of SOREM episodes in 5 infected rats sacrificed at different days post-infection (dpi) are shown on the left; the total number of parasites (*T*. *b*. *brucei*, *Tbb*) counted in the brain parenchyma at anterior and posterior levels (same levels as those shown in Fig 3) in each animal at the time of sacrifice is shown on the right (**C**). The height of the whole bar in A represents the total number of SOREM episodes, the white portion corresponds to those occurring during the day (the period of sleep dominance) and the black portion during the night. In B the black and white portions represent the SOREM episode duration in seconds for day and night respectively, while the height of the whole bar indicates the sum of the mean episode length during the day and night. PH, posterior hypothalamus;other Abbreviations as in the legend to Fig 3.

The animals in which SOREM events were analysed with continuous telemetric monitoring were sacrificed during the time period identified above as critical for parasite neuroinvasion (11 and 15 dpi) or later (19 and 21 dpi). The data obtained in this part of the study revealed intriguing results (Fig 5).

SOREM periods were already evident in the first week (4–7 dpi) in animals 1, 2 and 4 during both day and night (Fig 5A), and had a relatively long duration (Fig 5B). SOREM episodes were documented in all the five cases during the progression of the infection, increasing in number and duration from the second week (Fig 5A and 5B). The episodes occurred during daytime (corresponding to the period of sleep dominance in nocturnal rodents) and at night, with a definite prevalence during the day (Fig 5A). Most SOREM episodes were relatively long during both day and night and their duration showed a tendency towards increase during the progression of the infection (Fig 5B).

When parasite neuroinvasion was verified in the brain of the same animals (Fig 5C), the severity (number and duration) of SOREM episodes and density of parasites in the neuropil at sacrifice did not show a direct relationship. For example, animal 2 with a relatively high number of SOREM episodes (Fig 5A) exhibited a relatively low number of parasites in the neuropil (Fig 5C), while animals 1 and 3 with a lower number of SOREM episodes (Fig 5A) exhibited relatively high numbers of parasites in the neuropil (Fig 5C).

## Discussion

In a rat model of African trypanosomiasis with a course of approximately 35 days, the present observations demonstrate that parasite neuroinvasion begins days after the peripheral infection. The distribution of trypanosomes exhibited regional differences in the brain, lymphocyte recruitment showed similar timing and distribution, and correlated with parasite infiltration. Importantly, monitoring of body weight and sleep structure alterations together with the verification of parasites in the brain parenchyma of the same animals provided evidence of an early onset of functional changes.

### Parasites and lymphocytes infiltrate the neuropil over time

In the present study, parasites were detected in the brain by qPCR at 6 dpi. However, this technique cannot confirm the location of the parasites within the brain. Therefore these parasites could have been located within the choroid plexus, an area where, as mentioned previously, they have been detected prior to neuroinvasion [13, 42], or persisted in low numbers in blood vessels despite perfusion. Importantly, the qPCR data showed a significant increase in parasite load at 21 dpi.

Our analysis at the cellular level demonstrated that parasites do not invade the neuropil immediately following infection, as also indicated by previous studies on parasite transmigration of the BBB [43]. A similar time interval was also observed for detection of extravascular parasites in the meninges in a murine model of the infection using an intravital imaging approach [44]. It is possible that the immune response in the brain could, for a short period of time, control the incumbent parasite population. In our study, the density of parasites in the brain showed variability between individual animals, particularly in the second and third weeks of infection. This could reflect successive waves of parasitaemia, or other mechanisms. Cyclical appearance of the parasites has also been observed in the CSF, with about one day delay with respect to parasitaemia, after intrathecal infection, in a paradigm in which parasite crossing of the blood-CSF barrier in the choroid plexus has been hypothesised [42]. There is mounting evidence to suggest that the neuroinflammatory response is the key parameter for parasite neuroinvasion, as a dissociation between parasitaemia and severity of brain inflammation has been reported in murine models of the infection [45].

The co-occurrence of parasites and CD4+ and CD8+ T-cells is in line with data showing that parasite migration across the BBB requires interferon (IFN)-γ secreted from activated lymphocytes [12]. The infiltration of lymphocytes in the neuropil was in general less dense than that of parasites. On the other hand, magnetic resonance imaging in a murine model of the infection has indicated that the degree of BBB impairment can be more severe than inflammatory cell infiltration in the brain during the disease [18, 46].

The relatively high concentration of parasites and lymphocytes in the posterior hypothalamus seems to reflect different gradients of permeability of the BBB around the median eminence, a circumventricular organ, and the arcuate nucleus of the hypothalamus which is connected with the median eminence [47]. Even taking into account these gradients, our data indicate that the active process of parasite transmigration of the BBB [48] is required for parasite entry into the hypothalamus where parasites were visualized traversing blood vessel walls.

The present findings are at variance with previous observations based on the invasive approach of intravital microscopy in murine models of *T*. *b*. *brucei* and *T*. *b*. *rhodesiense* infection, in which parasites were seen to exit brain vessels within hours, but the occurrence of parasites in the neuropil was not verified histologically [21]. Our demonstration of trypanosomes in the brain parenchyma also contradicts recent findings utilising freeze fracture electron microscope which failed to detect parasites in the neuropil [19, 22]. However, such ultrastructural studies might have been hampered by the sampling restraints required for electron microscopy. The present observation suggests a bidirectional movement across cerebral microvessels, as previously observed in vitro in a BBB model [49], which would require confirmation based on in vivo observations.

Parasite invasion through the perivascular Virchow-Robin spaces, based also on novel findings on CSF circulation [41], has also been suggested [20].

### Signs of disease during the evolution of African trypanosomiasis

As far as we are aware this is the first report of parasite invasion of the neuropil in African trypanosomiasis analysed together with functional signs of disease. The relatively early alterations in body weight reported here are in agreement with data recorded in a more chronic model of *T*. *b*. *brucei* infection in rats [50]. However, our findings do not support the proposal that body weight alterations could act as a marker of second stage disease [25, 26]. This suggestion was derived from the observation of sudden body weight loss, preceded by a drop in food intake, around 12 dpi in rats with an average infection course of 2 weeks [26], and therefore in a more acute model than the present chronic infection. Different rat models of the infection could account for such disparity.

The present study also shows that sleep structure alterations can start early after infection. The finding that the number and duration of SOREM periods increase during disease progression extends and strengthens our previous study in *T*. *b*. *brucei*-infected rats, where SOREM episodes were noted but parasite neuroinvasion was not verified histologically [51]. The present study also demonstrates that the severity of these sleep alterations does not correlate with the parasite density in the neuropil at sacrifice.

Furthermore, our findings do not point to an association between initial parasite neuroinvasion and onset of SOREM periods. Thus, our data indicate that SOREM periods cannot provide a biomarker *per se* of second stage sleeping sickness, but a high frequency of these sleep changes is an indicator of this stage, as also proposed in cases of HAT on the basis of polysomnography [27]. In patients, SOREM periods can correspond to a rapid drop in neck muscle tone, and manifestation of this phenomenon could therefore be of clinical relevance.

In humans, SOREM episodes can be found in other conditions and are characteristic, together with other sleep-wake changes, of the chronic sleep disorder narcolepsy [52], and its murine models [53, 54]. Loss of orexin-containing neurons, which are located in the posterior lateral hypothalamus, characterizes human narcolepsy with cataplexy [55]. Findings from HAT patients [56] and infected rats [57] showed that the orexin level in the CSF is reduced in some cases but such a decrease is not consistent. Data in rodent models of the infection [57] point to loss and structural damage of a proportion of orexinergic neurons, but especially to severe dysfunction of these neurons, indicating that they are susceptible to inflammatory signalling, as previously reported [58].

High levels of pro-inflammatory cytokines, and in particular IFN-γ, tumor necrosis factor-α, interleukin-1ß, have been documented in the brain of *T*. *b*. *brucei*-infected rodents, and in the CSF and blood of HAT patients [59]. The disease is, therefore, dominated by inflammatory mediators and their critical balance. Inflammatory chemokines [60, 61], and in particular CXCL10, have been proposed to play a role in parasite neuroinvasion [12]. However, in Rhodesian HAT neurological symptoms and signs have not been found to be related to plasma and CSF immunoglobulin levels or CSF cytokine synthesis [34]. Our data suggest a regional susceptibility of the hypothalamus to early inflammatory responses, possibly related to early parasite invasion of circumventricular organs which could trigger early dysfunction of susceptible sleep-wake regulatory cell groups.

### Conclusion

Our findings show that parasites and T-cells penetrate the brain parenchyma over time following infection. However, a time interval after initial infection is required prior to this brain invasion. Importantly, functional disturbances indicative of brain involvement can occur during the initial phase of peripheral trypanosome infection. The data also show that neurological features may not result from the direct effect of parasites immediately after they enter the brain parenchyma, and, conversely, that parasite and T-cell entry in the neuropil may not represent the primary cause of neurological alterations since these events occur early following infection. The severity of functional disturbances, however, worsens considerably during the encephalitic stage. The findings question the validity of the presence and number of trypanosomes and T-cells in the CSF as disease stage biomarkers, and focus attention on the need for more accurate methods to establish infection stage and the importance of objective monitoring of clinical disease severity in HAT.

## Supporting Information

S1 Fig Regional distribution of the number of parasites, CD4+ and CD8+ T-cells in the neuropil at different days post-infection. Counts (mean number in 3 adjacent sections per level) of *Trypanosoma brucei brucei* (*Tbb*) and lymphocytes made in different regions at the brain anterior and posterior levels shown in Fig 3. Abbreviations: cc, corpus callosum; Cx, neocortex; Hp, hippocampus; BF, preoptic area and basal forebrain; Sp, septum; St, striatum; *Tbb*, *T*. *b*. *brucei*; Th, thalamus.
(TIF)
