# Supplementary figures and images for: Trypanosoma brucei Invasion and T-Cell Infiltration of the Brain Parenchyma in Experimental Sleeping Sickness: Timing and Correlation with Functional Changes
Source: PLoS Negl Trop Dis. 2016 Dec 21;10(12):e0005242. doi: 10.1371/journal.pntd.0005242 (PMC5217973; doi:10.1371/journal.pntd.0005242)

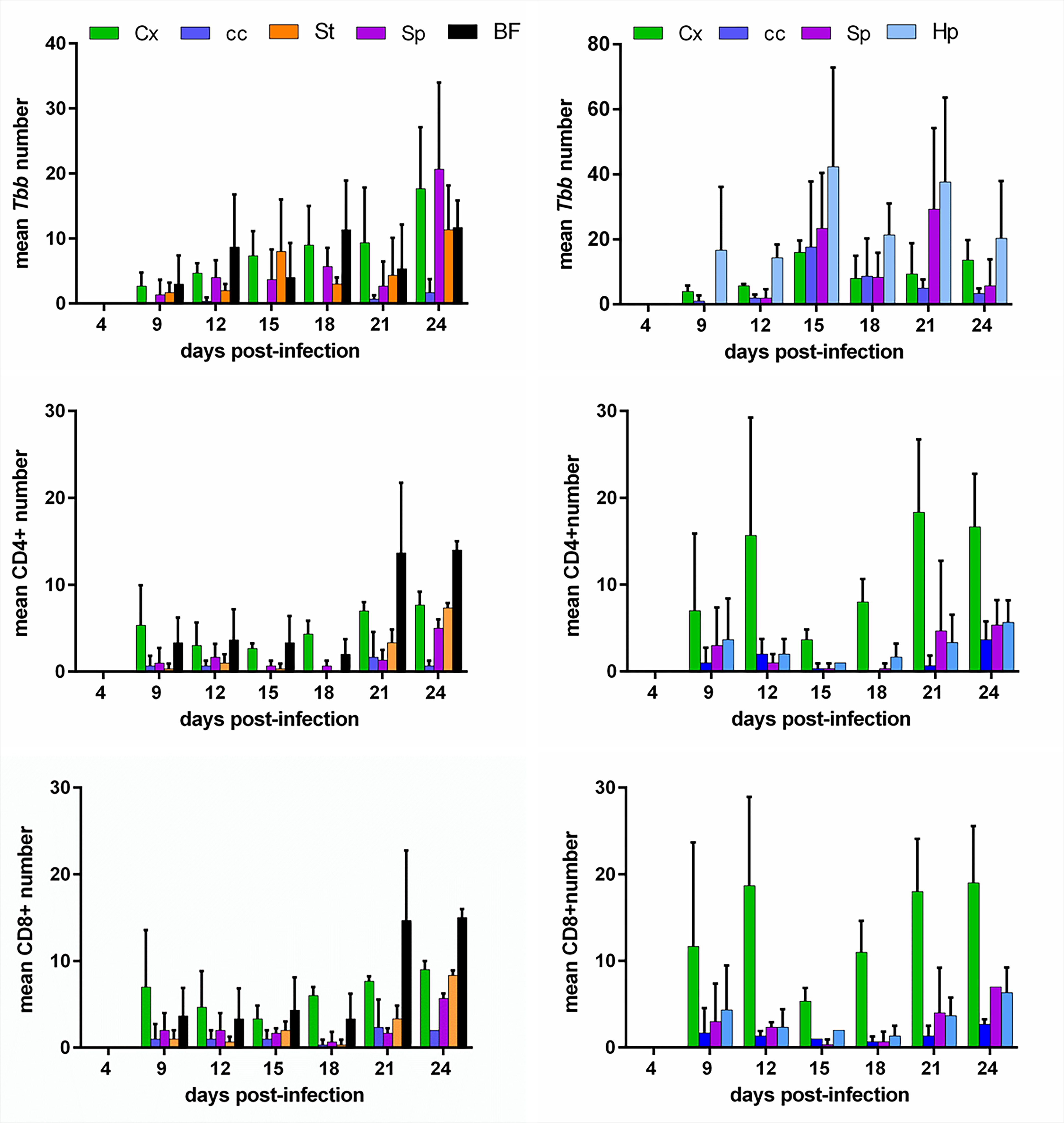

Supplement: S1 Fig — Counts (mean number in 3 adjacent sections per level) of Trypanosoma brucei brucei (Tbb) and lymphocytes made in different regions at the brain anterior and posterior levels shown in Fig 3. Abbreviations: cc, corpus callosum; Cx, neocortex; Hp, hippocampus; BF, preoptic area and basal forebrain; Sp, septum; St, striatum; Tbb, T. b. brucei; Th, thalamus. (TIF) [file pntd.0005242.s001.tif]
